# Mortality burden of bacterial antimicrobial resistance in East Africa: pooled analysis of modelled estimates

**DOI:** 10.1186/s41182-025-00870-x

**Published:** 2025-12-08

**Authors:** Yusuff Adebayo Adebisi, Najim Z. Alshahrani, Theogene Uwizeyimana

**Affiliations:** 1 Global Health Focus, Kigali, Rwanda; 2 College of Social Sciences, University of Glasgow, 40 Bute Gardens, Glasgow, G12 8RT UK; 3 Department of Family and Community Medicine, Faculty of Medicine, University of Jeddah, Jeddah, Saudi Arabia; 4 Bill and Joyce Cummings Institute of Global Health, University of Global Health Equity, Kigali, Rwanda

**Keywords:** Antimicrobial resistance (AMR), Age-standardised mortality rate (ASMR), East Africa, Bacterial infections, Surveillance

## Abstract

**Introduction:**

Bacterial antimicrobial resistance (AMR) is a major and growing public health threat in East African Community (EAC) countries, where fragile health systems, inadequate diagnostics, and inappropriate antibiotic use drive high levels of resistant infections. Despite this, robust subregional mortality estimates remain limited.

**Methods:**

We conducted a secondary pooled analysis of modelled, publicly available, country-level mortality estimates from the Global Research on Antimicrobial Resistance (GRAM) 2019 project. Data were extracted for six EAC countries: Burundi, Kenya, Rwanda, South Sudan, Tanzania, and Uganda. Because GRAM reports age-standardised mortality rates (ASMRs) with 95% uncertainty intervals, we derived standard errors from these intervals, assuming approximate normality, and used them in the pooled analysis. Using random-effects models with restricted maximum likelihood (REML), we pooled ASMRs per 100,000 population for deaths associated with and attributable to AMR. We calculated 95% confidence intervals and prediction intervals, assessed heterogeneity using I^2^, and conducted leave-one-out sensitivity analyses to test robustness.

**Results:**

Across the six EAC countries, there were an estimated 154,760 deaths associated with AMR and 36,480 deaths attributable to AMR in 2019. The pooled ASMR for AMR-associated deaths was 144.69 per 100,000 (95% CI 129.07–160.30) population, with a 95% prediction interval of 122.57–166.81. Country-specific ASMRs for AMR-associated deaths ranged from 129.5 per 100,000 population in Uganda to 167.0 per 100,000 population in Burundi. For AMR-attributable deaths, the pooled ASMR was 34.62 per 100,000 (95% CI 30.02–39.23) population, with a prediction interval of 28.10–41.14. Country-specific ASMRs for attributable deaths ranged from 30.80 per 100,000 population in Uganda to 41.90 per 100,000 population in Burundi. For both associated and attributable mortality, heterogeneity was negligible (I^2^ = 0%), and sensitivity analyses confirmed that no country disproportionately influenced the pooled estimates.

**Conclusion:**

This pooled secondary analysis indicates a substantial and regionally consistent mortality burden from bacterial AMR in East Africa. The findings reify the need for coordinated investment in AMR surveillance, stewardship, and overall response across the EAC.

**Supplementary Information:**

The online version contains supplementary material available at 10.1186/s41182-025-00870-x.

## Introduction

Antimicrobial resistance (AMR) poses a growing and multidimensional threat to global public health, with implications for the prevention and treatment of bacterial infections [1, 2]. The phenomenon occurs when pathogens evolve mechanisms that render antibiotics and other antimicrobial agents ineffective, resulting in higher morbidity, mortality, and healthcare costs [3]. Although AMR is a universal challenge, its consequences are especially severe in low- and middle-income regions, where health systems often lack the capacity to respond effectively [4]. In East Africa, the escalation of resistant infections undermines gains made in managing communicable diseases such as pneumonia, and tuberculosis, with resistance now documented even to last-resort antibiotics [5–8]. This crisis is further aggravated by widespread empiric antibiotic use, poor infection control practices, and the scarcity of microbiological diagnostics, creating a fertile environment for resistant pathogens to flourish undetected and unchecked [8–10].

A unique confluence of structural and health-system factors further amplifies the impact of AMR in the region [11–13]. These include high burdens of infectious diseases, considerable use of antibiotics in both human and animal health, and varying levels of access to clean water, sanitation, and healthcare services [14, 15]. Antibiotics are frequently dispensed over the counter without prescription, often in inappropriate dosages or for non-bacterial conditions, contributing to unnecessary selective pressure on microbes [16, 17]. Compounding this are weak regulatory frameworks and limited investment in laboratory infrastructure, which collectively hinder the identification and control of resistant infections. Although national AMR action plans have been adopted across several East African countries, their implementation is often constrained by financial, technical, and logistical challenges [18]. Surveillance remains patchy, fragmented, and under-resourced, resulting in significant data gaps that impede timely policy responses and regional coordination.

Recent global initiatives have sought to fill these knowledge gaps by producing modelled estimates of the burden of AMR, including the number of deaths directly attributable to resistant infections and those in which resistance played a contributing role. One of the most comprehensive of these is the Global Research on Antimicrobial Resistance (GRAM) project, which provides standardised, age-adjusted mortality estimates by pathogen and resistance type for 2019 [19]. These estimates are particularly valuable for regions like East Africa, where empirical data are scarce and often non-comparable across countries. However, despite the importance of these data, few studies have systematically synthesised them at a regional level in East Africa to quantify the mortality burden and guide context-appropriate policy interventions. Without a clear understanding of the scale and distribution of AMR-related deaths in the region, it remains difficult to prioritise investments in surveillance, stewardship, and health system strengthening.

This paper addresses this evidence gap by presenting a pooled analysis of antimicrobial resistance mortality across six East African Community (EAC) countries. Drawing on GRAM 2019 estimates, we examine age-standardised mortality rates per 100,000 population for deaths both associated with AMR (where resistance contributed) and attributable to AMR (where resistance was the direct cause). We also conduct leave-one-out sensitivity analyses to test the robustness of our findings and assess the influence of individual countries on pooled estimates.

## Methods

The GRAM study is the most comprehensive effort to date to estimate the global burden of bacterial AMR. Conducted by the Institute for Health Metrics and Evaluation in collaboration with the University of Oxford, GRAM systematically collated and modelled more than 470 million individual records and isolates from over 7,500 study-location-years across 204 countries and territories. Using a counterfactual framework, it generated harmonised estimates of deaths associated with AMR (where resistance contributed to the fatal outcome) and deaths attributable to AMR (the excess mortality caused by resistance compared with a susceptible counterfactual). These estimates provide directly comparable, policy-relevant country data suitable for regional pooling, and the methodology has been detailed elsewhere [19].

We extracted country-level estimates of deaths associated with and attributable to AMR from the GRAM 2019 supplementary document [19]. These data included age-standardised mortality rates (ASMRs) per 100,000 population, along with their corresponding 95% uncertainty intervals (UIs). We chose to use these modelled ASMRs rather than attempt to collate raw national data because empirical AMR data across East African countries are sparse, heterogeneous, and often non-comparable. By integrating diverse inputs such as surveillance systems, hospital-based reports, published literature, and vital registration where available, the GRAM framework produces harmonised estimates with UIs suitable for further analysis.

At present, there is no dedicated EQUATOR guideline for this specific study design. Accordingly, we adhered to the RECORD extension of STROBE where applicable, marking not applicable items, and we provided a GATHER-style transparency box to clarify data sources, transformations, and uncertainty handling. A completed RECORD checklist is presented in Supplementary Table 1.

For this analysis, we included six EAC countries: Burundi, Kenya, Rwanda, South Sudan, Tanzania, and Uganda [20]. Countries were included in this pooled analysis based on their formal membership in the EAC as of 2019 [20]. We extracted two key indicators: deaths associated with AMR, where resistance contributed to the fatal outcome, and deaths attributable to AMR, where the resistant infection was deemed the direct cause of death compared with a scenario of susceptible infection (see Supplementary Table 2). Data extraction was performed by one author (YAA), with independent review and verification by a second author (NZA); discrepancies were resolved by consensus. As the GRAM supplementary tables are openly available and clearly structured, the process was straightforward and reproducible. Only estimates related to bacterial AMR were considered, in line with the GRAM methodology, which focuses on bacterial pathogens and their resistance profiles.

As standard errors (SEs) were not provided in the GRAM documentation, we estimated them from the reported UIs for each ASMR using the formula SE = (Upper UI − Lower UI) ÷ (2 × 1.96), assuming approximate normality and symmetry of the interval [21]. This transformation enabled us to incorporate ASMRs into the meta-analysis framework. The derived SEs were used to weight each country’s estimate, such that narrower UIs contributed proportionally greater influence. Mortality counts are presented with their original UIs, while ASMRs are presented with 95% confidence intervals recalculated from the derived SEs.

We then conducted random-effects pooled analyses using the restricted maximum likelihood (REML) method to pool ASMRs across the six EAC countries. Analyses were performed separately for AMR-associated and AMR-attributable mortality. We declared each country’s ASMR and calculated standard error using the meta set command in Stata, followed by the meta summarize command to generate pooled estimates and 95% confidence intervals. We assessed statistical heterogeneity using the I^2^ statistic, tau-squared (τ^2^), and Cochran’s Q test. Forest plots were generated with the meta forestplot command to visually present individual country estimates, pooled estimates, and their respective confidence intervals. Additionally, we calculated 95% prediction intervals for both outcomes, reflecting the expected range of mortality rates that might be observed in a similar East African setting not included in this analysis. These prediction intervals enhance the generalisability of our findings and offer actionable insight for regional policy planning.

To test the robustness of our pooled estimates and identify potentially influential outliers, we conducted a leave-one-out sensitivity analysis. This involved systematically omitting one country at a time and re-running the random-effects analysis to evaluate how each exclusion influenced the overall result. This procedure, implemented using the leaveoneout option in Stata, helped to determine whether any single country disproportionately influenced the pooled ASMRs.

All analyses were performed using Stata/SE 18.0 (StataCorp, College Station, TX, USA).

## Results

**Fig. 1:**
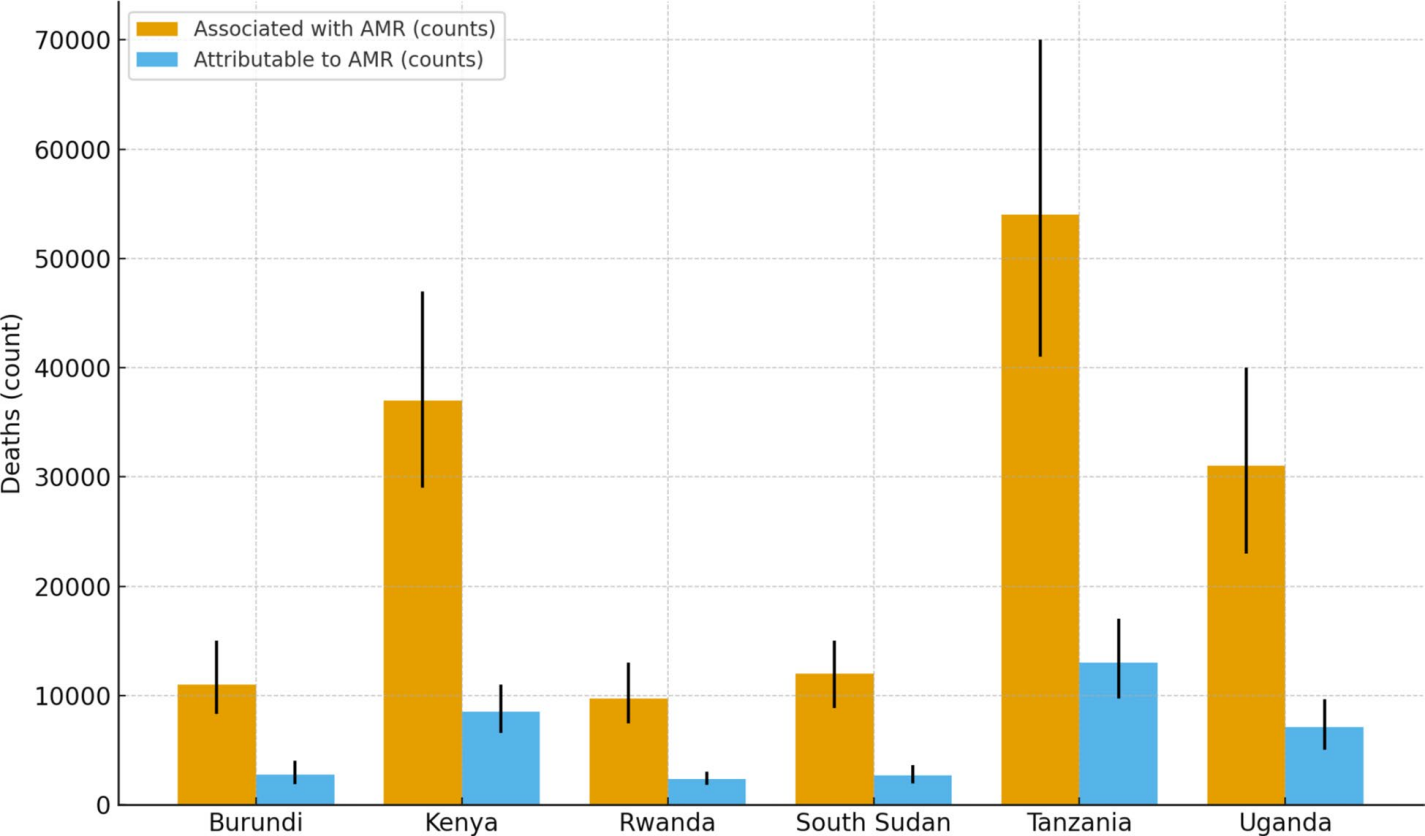
Bacterial AMR mortality counts in East African Community countries, 2019. Bars show central estimates with 95% uncertainty intervals. “Associated” refers to deaths in which resistance contributed to the outcome, whereas “attributable” refers to excess deaths directly caused by resistance compared with a susceptible infection counterfactual

Across the six EAC countries, the total number of deaths associated with bacterial AMR in 2019 was 154,760, while deaths directly attributable to AMR accounted for 36,480. Country-level burdens varied but remained consistently high. Tanzania reported the largest number of deaths associated with AMR, with a central estimate of 54,000 deaths and a 95% UI of 41,000–70,000, and attributable deaths of 13,000 (95% UI: 9,720–17,000). Kenya followed with 37,000 associated deaths (95% UI: 29,000–47,000) and 8,540 attributable deaths (95% UI: 6,570–11,000). Uganda reported 31,000 associated deaths (95% UI: 23,000–40,000) and 7,110 attributable deaths (95% UI: 5,070–9,660) (see Table 1 and Fig. 1).

**Table 1:** Deaths associated with and attributable to bacterial AMR in East African Community countries, 2019

Country	Deaths associated with AMR (counts)	Deaths attributable to AMR (counts)
Burundi	11,000 (8,340–15,000)	2,750 (1,910–4,050)
Kenya	37,000 (29,000–47,000)	8,540 (6,570–11,000)
Rwanda	9,760 (7,430–13,000)	2,370 (1,850–3,000)
South Sudan	12,000 (8,840–15,000)	2,710 (1,960–3,650)
Tanzania	54,000 (41,000–70,000)	13,000 (9,720–17,000)
Uganda	31,000 (23,000–40,000)	7,110 (5,070–9,660)

Values represent central estimates with 95% uncertainty intervals (lower–upper) as reported in the GRAM 2019 study [19]. “Associated” refers to deaths in which resistance contributed to the fatal outcome, whereas “attributable” refers to excess deaths directly caused by resistance compared with a susceptible infection counterfactual

### Age-standardised mortality rates associated with bacterial AMR in East Africa

**Fig. 2:**
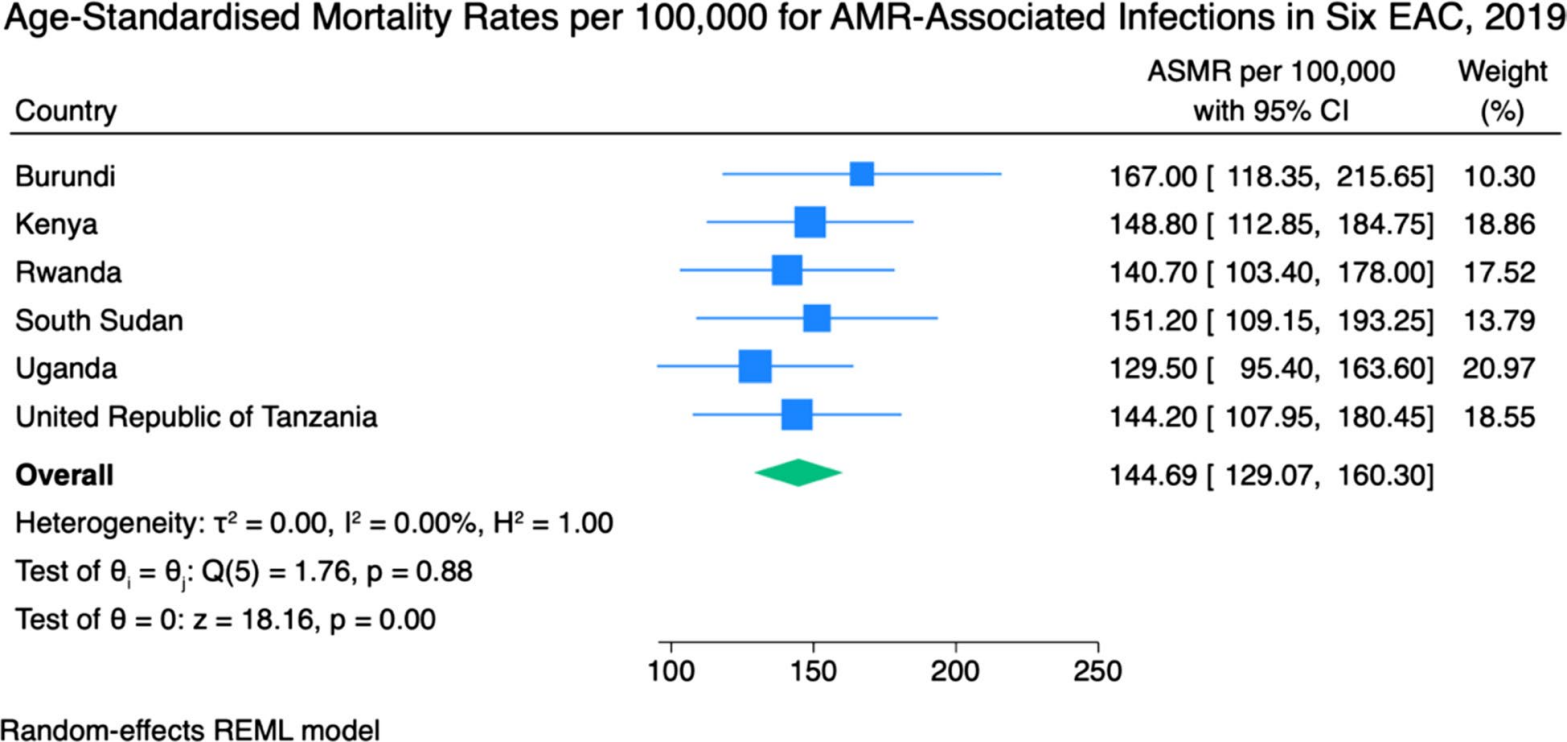
Age-standardised mortality rates per 100,000 population for deaths associated with antimicrobial resistance in six East African Community countries

Figure 2 presents the ASMRs per 100,000 population for deaths associated with bacterial antimicrobial resistance in six EAC countries. Country-specific estimates ranged from a low of 129.50 per 100,000 population in Uganda (95% CI 95.40–163.60) to a high of 167.00 per 100,000 population in Burundi (95% CI 118.35–215.65). The pooled ASMR across the region was 144.69 per 100,000 (95% CI 129.07–160.30) population. The 95% prediction interval for the pooled estimate was 122.57 to 166.81 per 100,000 population. Heterogeneity across countries was negligible, with I^2^ = 0.0%, τ^2^ = 0.000, and a non-significant Cochran’s Q statistic (Q = 1.76; p = 0.882).

### Age-standardised mortality attributable to bacterial AMR in East Africa

**Fig. 3:**
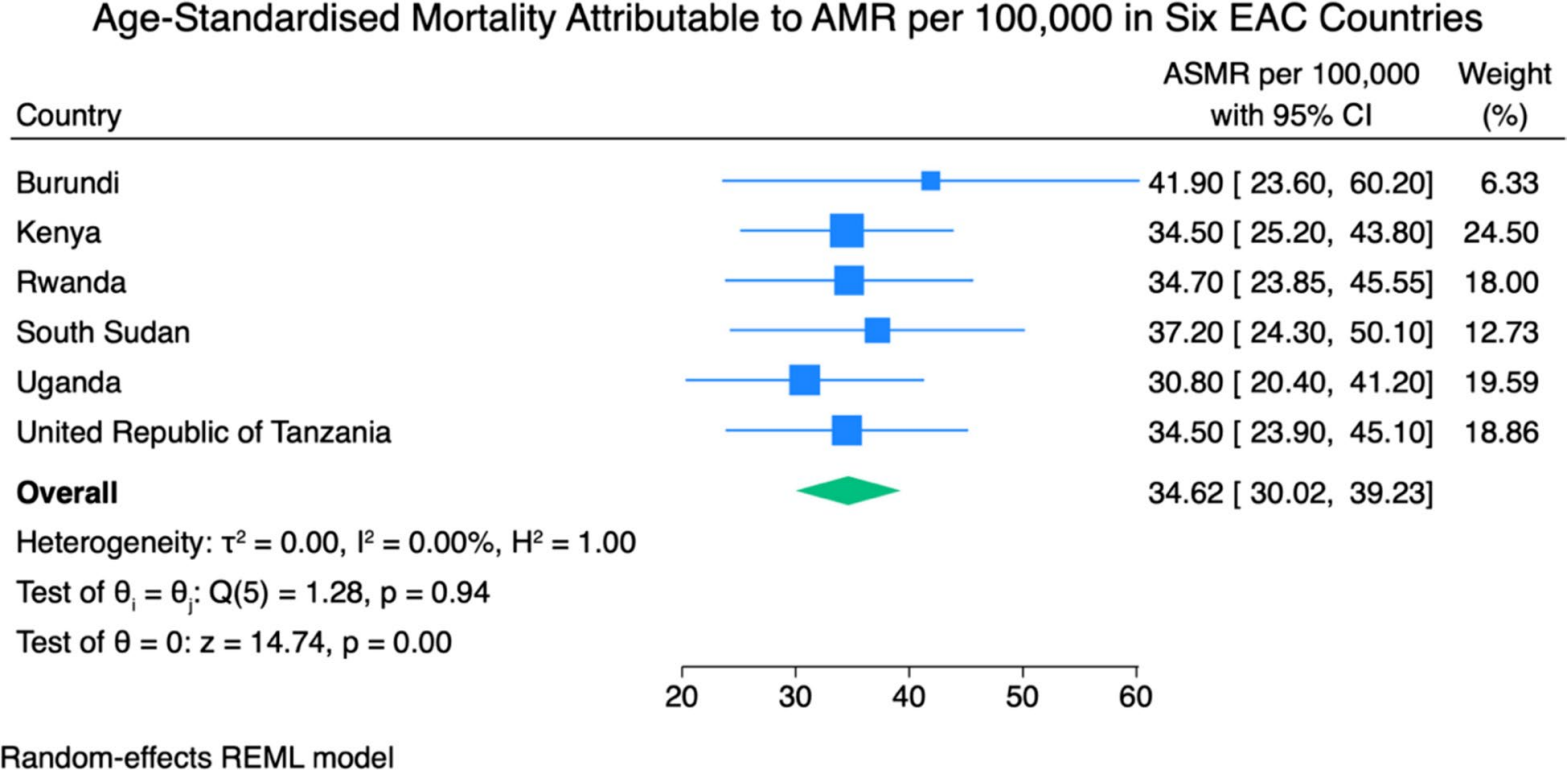
Age-standardised mortality attributable to bacterial AMR per 100,000 in six EAC countries

Figure 3 shows the ASMRs per 100,000 population for deaths attributable to antimicrobial resistance across the six EAC countries. The country-specific attributable ASMRs ranged from 30.80 per 100,000 population in Uganda (95% CI 20.40–41.20) to 41.90 per 100,000 population in Burundi (95% CI 23.60–60.20). The pooled ASMR for the region was 34.62 per 100,000 (95% CI 30.02–39.23) population. The 95% prediction interval was 28.10 to 41.14 per 100,000 population. As with the associated mortality analysis, heterogeneity was negligible (I^2^ = 0.0%, τ^2^ = 0.000), and the Cochran’s Q test was non-significant (Q = 1.28; p = 0.937), indicating strong consistency across national estimates.

### Sensitivity analysis

**Fig. 4:**
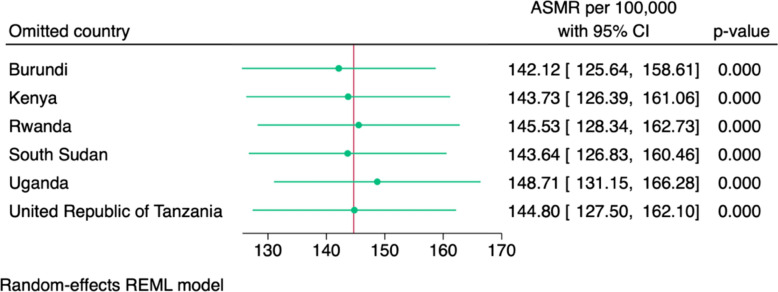
Leave-one-out sensitivity analysis of age-standardised mortality rates per 100,000 for deaths associated with antimicrobial resistance in six East African Community countries

Figure 4 displays the results of a leave-one-out sensitivity analysis for the pooled age-standardised mortality rate associated with antimicrobial resistance in the six East African Community countries. Each iteration of the analysis excluded one country at a time and re-estimated the pooled ASMR using a random-effects REML model. The recalculated estimates ranged from 142.1 per 100,000 (when Burundi was omitted) to 148.7 per 100,000 (when Uganda was excluded). All confidence intervals overlapped substantially with the original pooled estimate of 144.7 per 100,000.

**Fig. 5:**
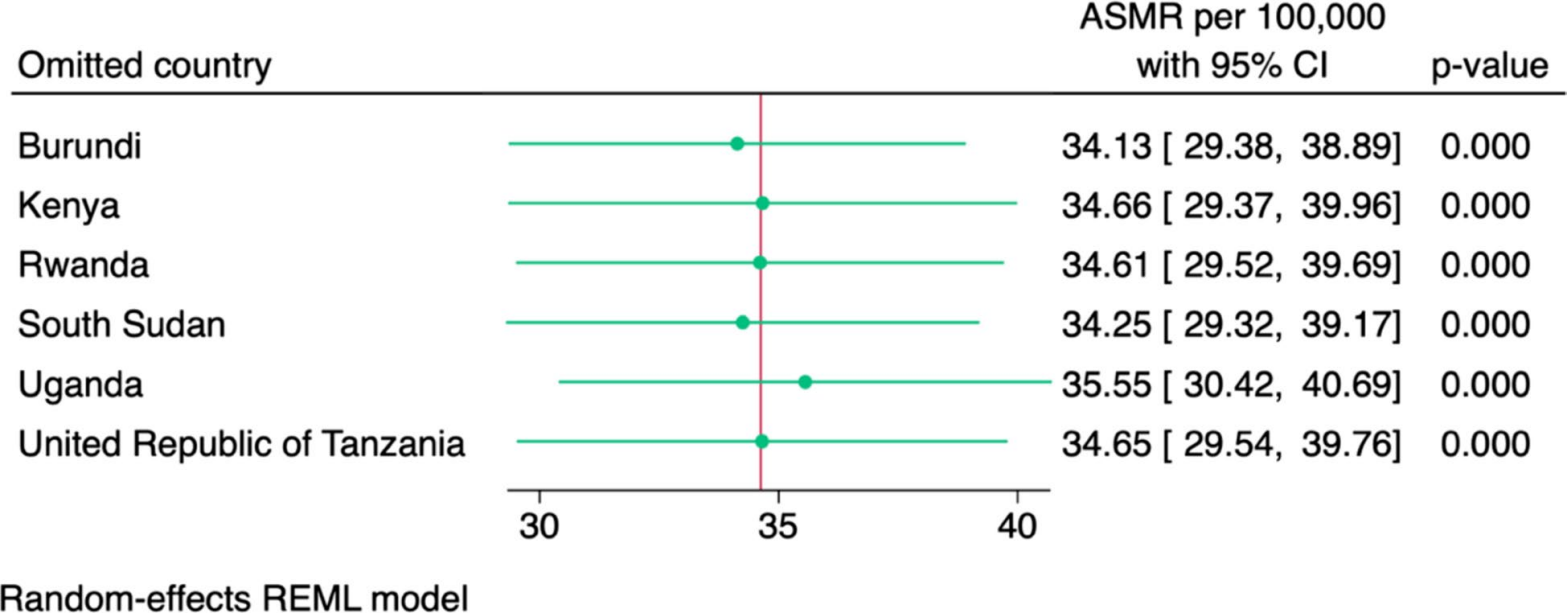
Leave-one-out sensitivity analysis of age-standardised mortality attributable to antimicrobial resistance per 100,000 in six East African Community countries, 2019

Figure 5 illustrates the leave-one-out sensitivity analysis for age-standardised mortality rates attributable to antimicrobial resistance in the six East African Community countries. Each analysis iteration omitted one country and recalculated the pooled estimate using a random-effects REML model. The resulting pooled ASMRs varied only slightly, ranging from 34.1 per 100,000 (excluding Burundi) to 35.6 per 100,000 (excluding Uganda). All 95% confidence intervals substantially overlapped with the main pooled estimate of 34.6 per 100,000.

## Discussion

This study presents the first pooled estimates of ASMRs associated with and attributable to antimicrobial-resistant bacterial infections across six EAC countries, using 2019 data from the GRAM project. The associated ASMR of 144.69 per 100,000 population and attributable ASMR of 34.62 per 100,000 population underline that AMR is not merely an emerging concern but a major driver of preventable deaths in East Africa. The true burden may be even higher, as weak vital registration systems, underdeveloped laboratory infrastructure, and limited access to microbiological testing mean that many AMR-related deaths go unrecorded [22]. By quantifying this mortality burden across countries with different health system capacities, our findings offer critical evidence for policy prioritisation and resource mobilisation. Moreover, the use of age-standardised rates ensures comparability across settings with varying population structures, allowing interventions to be targeted more equitably.

Compared to other leading causes of death in East Africa, AMR emerges as a comparable, if not more urgent, threat. For instance, in 2019 malaria was estimated to cause 56.3 deaths per 100,000 in sub-Saharan Africa [23], while HIV-related mortality stood at 45.5 per 100,000 in 2021 [24]. In contrast, our analysis shows that AMR-associated mortality alone reached nearly 145 per 100,000, placing it well above these high-priority conditions. Despite this burden, investment in AMR remains disproportionately low across East Africa [25]. Elevating AMR to a priority health threat on par with malaria and HIV is essential for securing long-term investments in diagnostics, stewardship, and infection prevention and control (IPC) programmes. Without these, the region will continue to face avoidable mortality and widening health inequities driven by resistant infections.

While our analysis found relatively consistent mortality rates across the six EAC countries, underlying drivers of AMR may differ. In urban Kenya or Tanzania, relatively better diagnostic capacity may allow higher detection of resistant infections, but unregulated antibiotic sales through private pharmacies still contribute to misuse [15, 16, 26]. In more fragile settings such as South Sudan, lack of infrastructure, prolonged conflict, and humanitarian crises may limit both treatment access and surveillance capacity, resulting in undiagnosed resistant infections and unchecked transmission. Across the region, widespread empirical antibiotic use, limited access to second-line treatments, and weak antimicrobial stewardship are major contributors [8, 12, 27]. Additional contextual factors, including poor sanitation, high population density, limited access to clean water, and inadequate waste management, exacerbate bacterial disease transmission and increase the reliance on ineffective antibiotics [26–28]. Climate variability, such as flooding in Lake Victoria Basin or drought-related displacement in pastoralist regions, may further alter infection dynamics and AMR patterns [29, 30]. Addressing AMR in East Africa therefore requires integrated, context-specific strategies that go beyond healthcare to include WASH (water, sanitation and hygiene), education, agricultural regulation, and cross-border coordination [25–28].

The 95% prediction intervals in this study (122.6–166.8 for associated ASMR; 28.1–41.1 for attributable ASMR) offer practical benchmarks for countries with sparse AMR surveillance, providing realistic mortality ranges to inform planning and target-setting. Regional agencies like the EAC Secretariat and Africa CDC could use these benchmarks to promote data sharing, develop harmonised surveillance protocols, and support capacity-building initiatives across member states. At the national level, health ministries could incorporate these estimates into AMR action plans and set measurable mortality reduction targets, such as a 10–15% decline in AMR-associated deaths over 5 years, tracked through sentinel surveillance or facility-based IPC programmes. Furthermore, communicating the prediction intervals alongside point estimates can help advocate for AMR control with appropriate nuance: even the lower bound of the range represents a significant mortality burden. This dual messaging strategy, emphasising urgency but acknowledging uncertainty, may be especially useful in engaging ministries of finance, donors, and other non-health actors who influence national budget priorities.

Our findings should also be considered within the broader challenges of AMR surveillance in Africa, particularly in East African and other low- and middle-income countries. Across much of the region, microbiological infrastructure remains weak, surveillance systems are fragmented, and vital registration is incomplete, resulting in major blind spots in understanding the true burden of drug-resistant infections [31–34]. These structural gaps mean that locally observed data are often sparse or non-comparable, leaving modelling initiatives such as the GRAM project as the only harmonised source of country-level and regional estimates. A commentary highlighted these same constraints, emphasising that the reliance on modelled estimates reflects the paucity and unevenness of empirical data, rather than methodological preference [35]. In East Africa, these challenges are compounded by high burdens of infectious diseases, widespread empiric antibiotic use, and weak stewardship mechanisms, which together heighten the mortality impact of AMR but remain poorly captured in routine health information systems [36–39]. In this context, our pooled analysis provides an important regional benchmark, offering synthesised and comparable mortality estimates while acknowledging that the true burden may be even greater in countries where under-detection and under-reporting are most severe. Pooling country-level ASMRs from GRAM is therefore a scientifically justified strategy to generate a consistent regional picture, while recognising the inherent limitations of modelled data.

Despite the rigour of this analysis, several limitations should be acknowledged. First, the estimates rely on modelled data from the GRAM project, which may introduce bias in settings with sparse primary data or non-standard reporting. Second, the back-calculation of standard errors from 95% uncertainty intervals assumes normal distribution, which may not hold in countries with highly skewed data or small populations. Third, this study represents a cross-sectional analysis of 2019 only and does not account for trends over time or the impact of major events such as the COVID-19 pandemic, which has likely affected AMR dynamics through changes in antibiotic prescribing [40–43]. Fourth, the analysis does not differentiate between pathogen- or drug-specific mortality burdens, which are critical for prioritising pathogen-specific responses. Fifth, pooling at the national level may obscure important subnational heterogeneity, such as urban–rural differences or disparities between stable and conflict-affected regions. Finally, the uncertainty intervals provided by GRAM may not fully reflect the true variability in data-scarce settings, potentially leading to over-confidence in some estimates.

Building on these findings, future research should prioritise the generation of high-quality, country-level and subnational data on AMR to complement modelled estimates. Strengthening routine laboratory surveillance and expanding sentinel site networks would improve the empirical basis for mortality estimates and allow monitoring of trends over time [44–46]. Linking AMR surveillance with clinical outcome data, including treatment failure and sepsis-related mortality, would provide a more complete picture of the health impact of resistance. Comparative analyses that integrate economic and social costs of AMR, particularly productivity losses and household-level financial strain, would also be valuable for informing investment priorities. Finally, the development of harmonised regional platforms for data sharing within the East African Community could enhance collective action, enabling more timely detection of resistance patterns and more effective regional responses.

## Conclusion

This study provides the first regionally pooled estimates of age-standardised mortality associated with and attributable to antimicrobial resistance across East Africa, using harmonised 2019 data from the GRAM project. The findings reveal a substantial and consistent burden, with AMR-associated mortality exceeding that of many well-funded global health priorities. The negligible heterogeneity and robust sensitivity analyses suggest a region-wide health challenge that demands urgent, coordinated action. To reduce avoidable deaths and prevent the further spread of resistance, East African countries must invest in AMR surveillance systems, strengthen antimicrobial stewardship, and ensure equitable access to effective antibiotics. These estimates can serve as a regional benchmark for monitoring progress and mobilising political commitment, both within countries and through collaborative platforms such as the East African Community and Africa CDC.

## Supplementary Information

Supplementary material 1.

## Data Availability

The data analyzed in this study were extracted from the publicly available Global Research on Antimicrobial Resistance (GRAM) 2019 dataset.
