# Supplementary material for: Mortality burden of bacterial antimicrobial resistance in East Africa: pooled analysis of modelled estimates
Source: Trop Med Health. 2025 Dec 8;53:181. doi: 10.1186/s41182-025-00870-x (PMC12683851; doi:10.1186/s41182-025-00870-x)
Supplement: Supplementary file 1 — Supplementary material 1. [file 41182_2025_870_MOESM1_ESM.pdf]

*Supplementary Material*

# Mortality Burden of Bacterial Antimicrobial Resistance in East Africa: Pooled Analysis of Modelled Estimates

Yusuff Adebayo Adebisi[1,2] Najim Z Alshahrani[3] Theogene Uwizeyimana[4]

## Supplementary Table 1. Adapted RECORD Checklist for Reporting Secondary Analyses

| Section/Topic | Item | STROBE/RECORD Requirement | Where in manuscript | Notes/Adaptation for this study |
|---|---|---|---|---|
| **Title and abstract** | 1 | Indicate the study's design with commonly used term(s). | Title, Abstract | Title specifies *"pooled analysis"* and Abstract clarifies it is a secondary analysis of GRAM 2019 estimates. |
| **Introduction: Background/rationale** | 2 | Explain scientific background and rationale. | Introduction | Context of AMR burden in East Africa given, rationale for pooling estimates stated. |
| **Objectives** | 3 | State specific objectives. | Introduction (last paragraph) | Aim: pooled analysis of AMR-associated and attributable mortality in EAC countries. |
| **Methods: Study design** | 4 | Present key elements of study design. RECORD 4.1: Specify type of data used, database name, geographic region, timeframe, and reference the original study. | Methods | Secondary analysis of GRAM 2019 estimates, sourced from Antimicrobial Resistance Collaborators. |
| **Setting** | 5 | Describe setting, locations, relevant dates. | Methods | Six EAC countries, data year = 2019. |
| **Participants** | 6 | For cohort/case-control/cross-sectional designs: eligibility, sources, methods. RECORD 6.1: If linkage used, describe methods. | Not applicable | No individuals; analytic unit = country. No record linkage performed. |
| **Variables** | 7 | Clearly define outcomes, exposures, predictors. | Methods | Outcomes = age-standardised mortality rates (ASMRs) associated with and attributable to AMR. No exposures/predictors included. |
| **Data sources/measurement** | 8 | Sources of data and assessment methods. RECORD 8.1: Describe database origin, population covered, linkage, validation. | Methods | Data extracted from GRAM 2019 modelled dataset. Full methods published in Lancet Global Health 2024. |

| Section/Topic | Item | STROBE/RECORD Requirement | Where in manuscript | Notes/Adaptation for this study |
|---|---|---|---|---|
| **Bias** | 9 | Address potential sources of bias. | Discussion | Limitations include reliance on modelled estimates, back-calculated SEs, no pathogen-specific breakdown. |
| **Study size** | 10 | Explain how study size was arrived at. | Methods | Six EAC countries included (based on formal membership as of 2019). |
| **Quantitative variables** | 11 | Explain how quantitative variables were handled. | Methods | ASMRs analysed per 100,000 population. SEs estimated from uncertainty intervals. |
| **Statistical methods** | 12 | Describe all methods, subgroups, missing data, sensitivity analyses. | Methods | Random-effects REML meta-analysis, heterogeneity tests, prediction intervals, leave-one-out sensitivity. |
| **Results: Participants** | 13 | Numbers at each stage. RECORD 13.1: If linkage used, describe. | Results | Six countries included; no exclusions. No linkage performed. |
| **Descriptive data** | 14 | Characteristics of study sample. | Results | Country-specific ASMRs reported. |
| **Outcome data** | 15 | Report outcomes. | Results | Country-specific and pooled ASMRs reported with CIs and prediction intervals. |
| **Main results** | 16 | Give unadjusted and adjusted estimates. | Results | Pooled estimates reported; no adjustments beyond random-effects model. |
| **Other analyses** | 17 | Report subgroup and sensitivity analyses. | Results | Leave-one-out sensitivity analyses performed. |
| **Discussion: Key results** | 18 | Summarise key results. | Discussion | AMR mortality burden exceeds malaria/HIV mortality in region. |
| **Limitations** | 19 | Discuss limitations of study. RECORD 19.1: Discuss implications of using secondary data. | Discussion | Reliance on modelled GRAM data, lack of pathogen specificity, back-calculation of SEs. |
| **Interpretation** | 20 | Provide cautious interpretation. | Discussion | Interpretation given, comparisons with other diseases, policy implications. |
| **Generalisability** | 21 | Discuss external validity. | Discussion | Prediction intervals provide regional benchmarks. |
| **Other information: Funding** | 22 | Sources of funding. | Declarations | No specific funding for this analysis. |

**Supplementary Table 2. Variables extracted from the GRAM 2019 dataset for pooled analysis**

| Category | Variable | Description |
|---|---|---|
| **Outcome indicators** | ASMR – associated with bacterial AMR | Age-standardised mortality rate (per 100,000 population) for deaths where resistance contributed to the fatal outcome. Absolute counts were extracted too. |
| | ASMR – attributable to bacterial AMR | Age-standardised mortality rate (per 100,000 population) for deaths directly caused by resistance, compared with a susceptible infection counterfactual. Absolute counts were extracted too. |
| **Uncertainty measures** | 95% uncertainty interval (UI) | Lower and upper bounds of the 95% uncertainty interval for each ASMR, as reported in GRAM. |
| **Geographic identifiers** | Country | Six East African Community countries: Burundi, Kenya, Rwanda, South Sudan, Tanzania, Uganda. |
| **Temporal identifiers** | Year | Estimates restricted to the reference year 2019. |
